# Colorectal cancer-specific microbiome in peripheral circulation and cancer tissues

**DOI:** 10.3389/fmicb.2024.1422536

**Published:** 2024-08-21

**Authors:** Shushan Yan, Tie Liu, Haobin Zhao, Chunbo Zhao, Yuxin Zhu, Wenqing Dai, Wenchang Sun, Honggang Wang, Junxi Sun, Lu Zhao, Donghua Xu

**Affiliations:** ^1^Department of Gastrointestinal and Anal Diseases Surgery, Affiliated Hospital of Shandong Second Medical University, Weifang, China; ^2^Department of Microbiology and Immunology, Tulane University School of Medicine, New Orleans, LA, United States; ^3^Department of Anorectal Surgery, Weifang People's Hospital, Shandong Second Medical University, Weifang, China; ^4^Central Laboratory, Weifang People's Hospital, Shandong Second Medical University, Weifang, China; ^5^Clinical Laboratory, Weifang People's Hospital, Shandong Second Medical University, Weifang, China; ^6^Department of Biostatistics, School of Public Health, Cheeloo College of Medicine, Shandong University, Jinan, China; ^7^Shandong Laibo Biotechnology Co., Ltd., Jinan, China; ^8^Department of Rheumatology and Immunology, Weifang People's Hospital, Shandong Second Medical University, Weifang, China

**Keywords:** colorectal cancer, circulation and tissue, microbiome, microbial diversity, signaling pathway

## Abstract

**Introduction:**

Accumulating evidence has supported that gut microbiota and metabolite profiles play indispensable roles in the pathogenesis of colorectal cancer (CRC), which ranks as the third most common cancer and the second leading cause of cancer-related deaths worldwide. However, alterations in tumoral or circulating microbiomes in CRC remain incompletely understood. It has been well-documented that tissue or serum microbiomes with low microbial biomass could be screened by use of 2bRAD sequencing for microbiome (2bRAD-M) at the species resolution.

**Methods:**

In order to validate the microbial biomarkers distinguishing CRC and the variations in microorganisms present in serum and tumors, we performed 2bRAD-M to characterize the microbiomes in serum and cancer tissues of CRC patients with and without lymph node or liver metastasis.

**Results:**

The composition of dominated microbiota in serum was different from that of tissue samples, while the microbial community composition of tumors was similar to that of the tumor-adjacent tissues. The analysis of α-diversity and β-diversity has revealed notable variations in serum microbiota diversities in CRC patients, particularly those with liver metastasis. Multiple CRC-specific microbial species, such as Moraxella A cinereus, Flavobacterium sp001800905, and *Acinetobacter albensis*, were identified in serum. Complicated functions and KEGG pathways were also confirmed in CRC according to the metastasis status.

**Discussion:**

This study has found significant alterations in the microbial compositions and diversities in CRC and CRC-specific microbial species in both circulation and cancer tissues, which may serve as promising biomarkers for the screening, diagnosis and prognosis prediction of CRC. In particular, CRC-specific bacterial taxa are promising markers, holding transformative potentials in establishing personalized screening and risk stratification, refining much earlier non-invasive diagnostic approaches, and enhancing diagnostic sensitivity.

## 1 Introduction

Colorectal cancer (CRC) is one of the most prevalent malignant tumors in the digestive system, which is the third most commonly diagnosed cancer and the second leading cause of cancer death worldwide (Sung et al., 2021). According to the latest statistics of cancer incidence and mortality in China, the incidence and mortality of CRC are still increasing with 592,232 new diagnosed CRC cases and 56,693 CRC deaths in 2022 (Wei et al., 2020; Xia et al., 2022). Despite significant strides in understanding the etiology and advanced treatment strategies, CRC continues to exact a heavy burden on public health. Further investigations are warranted to provide insight into the etiology and pathogenesis of CRC. Early screening and effective cancer prevention measures are also critical for global CRC control, particularly in China.

CRC is a disease attributed to multiple factors, such as genetic mutations, immune dysregulation and diet factors (Johnson et al., 2013; Vogelstein et al., 2013; Shen et al., 2022). Apart from genetic and immune system factors, a great body of studies have implicated a close link between microbiome and CRC (Song et al., 2019; Wang and Li, 2022). Increasing evidence has unveiled the pivotal role of microbiota dysbiosis, namely the disruption in microbial composition and function, in driving colorectal carcinogenesis. CRC-specific microbiota in circulation or cancer tissues are potential contributors to CRC. Accumulating investigations have spotlighted microbial biomarkers, including specific bacterial taxa and metabolites, as promising non-invasive tools for CRC screening and risk stratification. Previous studies have documented well that the community structure of gut microbiota influences the initiation and progression of CRC. For instance, the abundance of *Helicobacter pylori, Fusobacterium nucleatum, Escherichia coli*, and *Bacteroides fragilis* were increased, while *Faecalibacterium, Roseburia*, and *Bifidobacterium* were decreased in CRC patients (Wang et al., 2012; Gao et al., 2015; Gagnière et al., 2016; Koyande et al., 2022). The changes in the structure of gut microbiota and the bacterial metabolites can affect the balance of intestinal microenvironment by regulating the intestinal epithelial barrier integrity, inflammation, metabolic processes, and immune response (Arrieta et al., 2006; Bischoff et al., 2014; Yang et al., 2021). Increased intestinal permeability leads to enhanced pathogenic bacteria infringement into the circulation. Moreover, breakdown of the gut homeostasis due to imbalance of gut microbiota and immunity, results in carcinogenesis.

The earlier viewpoint was that the blood is sterile in healthy individuals (Castillo et al., 2019); however, updated findings have suggested that the bacterial DNA is present not only in blood samples from patients, but also healthy human blood (Potgieter et al., 2015; Schierwagen et al., 2019; Lawrence et al., 2022; Merino-Ribas et al., 2022). Disease-specific microbiome in circulation is associated with the initiation and progression of various diseases, such as atherosclerosis, cardiovascular disease, ischemic stroke, chronic kidney disease, liver fibrosis, and cancer (Potgieter et al., 2015; Schierwagen et al., 2019; Lawrence et al., 2022; Merino-Ribas et al., 2022). Hence, the circulating microbiome may be a novel marker for cancer diagnosis. Nevertheless, little is known about CRC-specific microbiome in circulation and intestinal tissues. In this study, we investigated the alterations of blood- and tissue-specific microbiota at the species level in CRC with and without lymph node or liver metastasis by use of 2bRAD sequencing for microbiome (2bRAD-M), aiming to navigate the multifaceted interplay between microbiota and CRC, with a focus on the implications for CRC diagnosis, treatment efficacy, and prognostic assessment. Unraveling the microbial signatures associated with CRC is highly significant, as it has the potential to transform diagnostic approaches, improve sensitivity, and introduce personalized screening protocols.

## 2 Materials and methods

### 2.1 Study design and sample collection

This case-control study enrolled 23 adult CRC patients, including six CRC patients without liver metastasis and three CRC patients with liver metastasis for microbiota sequencing, and the serum from the remaining 14 CRC patients for validation experiments. Patients were admitted at the Department of Anorectal Surgery of the First Affiliated Hospital and the Affiliated Hospital, Shandong Second Medical University, from January 2022 to July 2022. In total, 25 healthy controls were recruited for physical examination at the same time period, and three healthy control samples for microbiota sequencing, and the remaining 22 healthy controls for validation experiments. This study was approved by the Ethics Committee of the Affiliated Hospital, Shandong Second Medical University (2020YX011), which was performed in compliance with the Declaration of Helsinki. Written informed consent was obtained from all participants. Demographic and clinical information were collected from study participants at recruitment. Cancer stage classification was based on the TNM Staging system (American Joint Committee on Cancer, 2017). For CRC patients, the tumor and tumor-adjacent tissue were collected aseptically from the excised colon. Four milliliter of fresh peripheral venous blood was collected in a blank (without anticoagulant) tube and centrifuged at 3,000 rpm for 10 min to isolate the serum fraction. All samples were stored in −80°C until further experiments.

### 2.2 DNA extraction, library construction, and sequencing

Samples were processed for DNA extraction using the TIANamp Micro DNA Kit (Tiangen) following the manufacturer's instructions. 2bRAD-M libraries were constructed according to previous studies (Hong et al., 2022; Sun et al., 2022). In brief, genomic DNA was digested with restriction enzyme at 37°C for 3 h, and then ligation reaction with library-specific adaptors was performed. Polymerase chain reaction (PCR) was carried out at the following conditions, 16–28 cycles at 98°C for 5 s, 60°C for 20 s, 72°C for 10 s, and final extension of 10 min at 72°C. The resulting libraries were purified with the QIAquick PCR purification kit (Qiagen, Valencia, CA) and sequenced on Illumina Novaseq PE150 platform. Library construction and sequencing were performed in OE BioTech Co., Ltd. All sequencing raw data was deposited into NODE (accession No. OEP004048), and can be accessed at http://www.biosino.org/node.

### 2.3 2bRAD-M sequencing data processing

Raw data were filtered to extract the sequences containing enzyme site fragment, called “enzyme reads”. Clean reads were obtained from enzyme reads with the following criteria: (1) removing reads with >8% unknown bases; (2) removing reads containing more than 20% of low-quality bases (*Q*-value ≤ 30). Taxonomic profiles were performed using the 2bRAD-M computational pipeline: GitHub: https://github.com/shihuang047/2bRAD-M. Clean reads were mapped against the 2bRAD-M tag database (Sun et al., 2022) and get the annotation information of microbial taxa, and then estimate the relative abundance of each taxon in a sample.

Alpha diversity (Chao1, Shannon, and Simpson) was generated based on the relative abundance of taxonomic profiles using the R package vegan. Beta diversity was assessed by binary-jaccard distance algorithms and the results were visualized by principal coordinate analysis (PCoA). The differential taxa between groups were performed by linear discriminant analysis (LDA) effect size (LEfSe) analysis (Segata et al., 2011), and the threshold on the LDA score for discriminative features was 2.0. The prediction of the functional composition of metagenomes was based on marked gene sequence in KEGG database using phylogenetic investigation of communities by reconstruction of unobserved states 2 (PICRUSt2) software (Douglas et al., 2020).

### 2.4 Taurine ELISA assay

Two milliliter of fresh peripheral venous blood was collected in a blank (without anticoagulant) tube and centrifuged at 3,000 rpm for 10 min to isolate the serum fraction. The taurine level of serum was measured using the ELISA kits according to the manufacturer's protocol (COIBO BIO, Shanghai, China). Inter and intra-assay coefficient of variations for taurine were < 15%.

### 2.5 Statistical analysis

The results from collected data were expressed as mean ± SD. Statistical analysis was performed with R software (version 3.4.2) and GraphPad Prism software (version 9.0.0). For groups of comparation of microbial community, the Mann-Whitney test was used to evaluate the significant difference. Wilcox test was used to calculate the *P-*value of alpha diversity indices. Binary jaccard distance was conducted by permutational multivariate analysis of variance (PERMANOVA) to evaluate differences and the Kruskal Wallis test was used to assess the differential bacteria between the two groups. *P* < 0.05 was considered statistically significant difference.

## 3 Results

### 3.1 The clinical and demographic characteristics of participants

Six CRC patients without liver metastasis, three CRC patients with liver metastasis, and three healthy controls were recruited into the present study for sequencing. The clinical and demographic characteristics of the enrolled participants were listed in Table 1. The basic information of 14 CRC patients and 22 healthy controls for validation were listed in Supplementary Table S1.

**Table 1:** The clinical and demographic characteristics of all participants.

**Characteristics**	**CRC without liver metastasis (*n* = 6)**	**CRC with liver metastasis (*n* = 3)**	**Control (*n* = 3)**
Age	68.2 ± 9.3	69.7 ± 9.1	71.3 ± 4.0
Male	4 (66.7%)	0 (0%)	0 (0%)
Female	2 (33.3%)	3 (100%)	3 (100%)
Alcohol intake	1 (16.7%)	0 (0%)	0 (0%)
Smoking status	2 (33.3%)	0 (0%)	0 (0%)
TNM stage	II (3), III (3)	IV (3)	–
Lymph node metastasis	3 (50%)	0 (0%)	–
Antibiotics use (3 months before sampling)	0 (0%)	0 (0%)	0 (0%)

There is no statistical significance for age. The statistical significance for gender, alcohol intake, smoking status, TNM stage, and lymph node metastasis is not available, respectively.

### 3.2 Microbial compositions in serum samples and colorectal tissues

**Figure 1:**
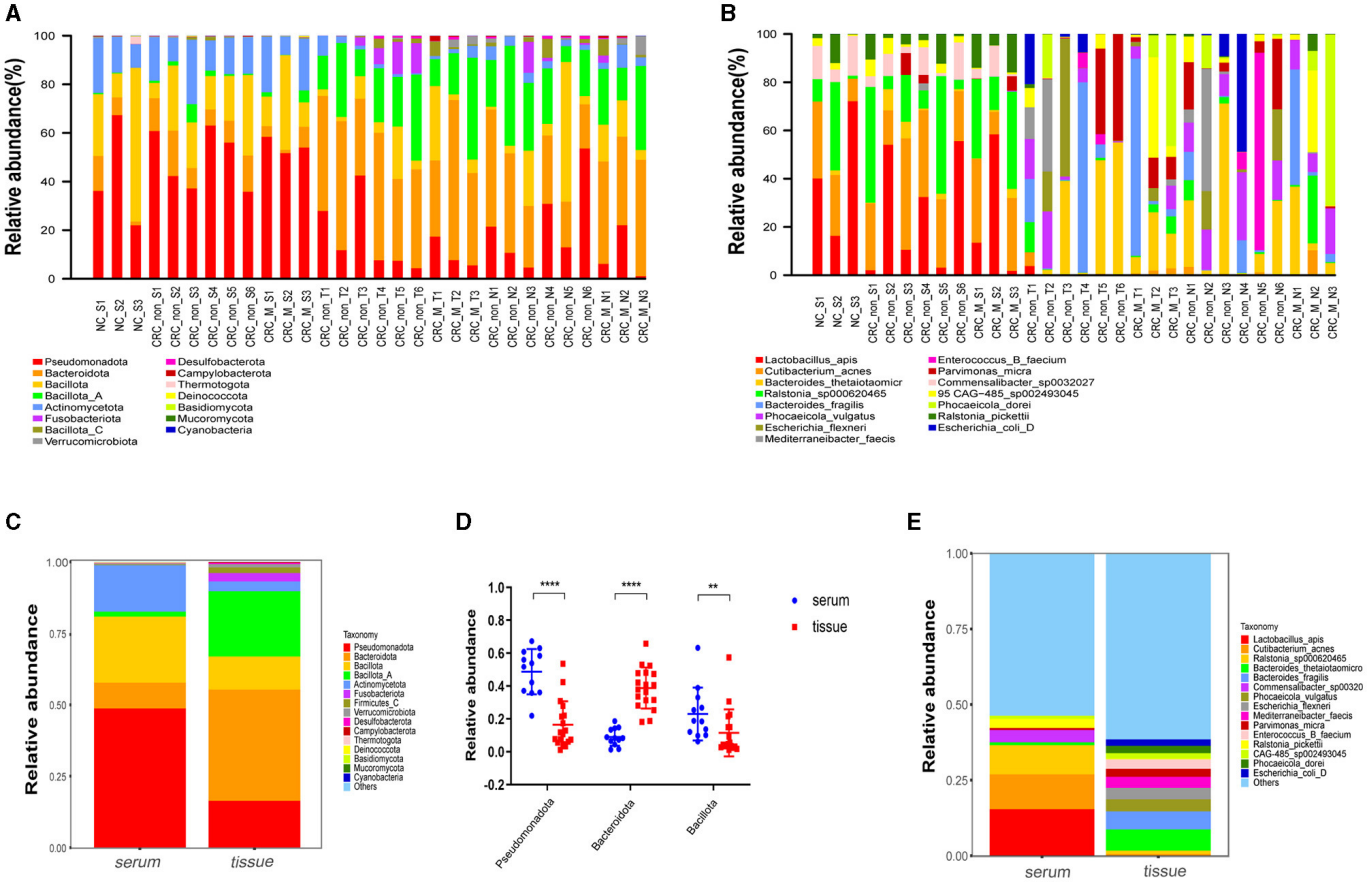
Microbial compositions in serum samples and colorectal tissues. **(A)** Relative abundance percentage of top 15 bacterial community in serum sample and colorectal tissues at phylum. **(B)** Relative abundance percentage of top 15 bacterial community of serum sample and colorectal tissues at species levels. NC_S, serum of healthy control; CRC_non_S, serum of CRC without liver metastasis; CRC_M_S, serum of CRC with liver metastasis; CRC_non_T, tumor of CRC without liver metastasis; CRC_M_T, tumor of CRC with liver metastasis; CRC_non_N, tumor-adjacent of CRC without liver metastasis; CRC_M_N, tumor-adjacent of CRC with liver metastasis. **(C)** Relative abundance of the bacteria at phylum levels in serum and tissue samples groups; **(D)** relative abundance of three dominated phylum bacteria in serum and colorectal tissues sample groups, Mann-Whitney test is performed to determine the differences between serum and tissue groups. **P < 0.01, ****P < 0.0001. **(E)** Relative abundance of the bacteria at species levels in serum and tissue samples groups.

**Figure 2:**
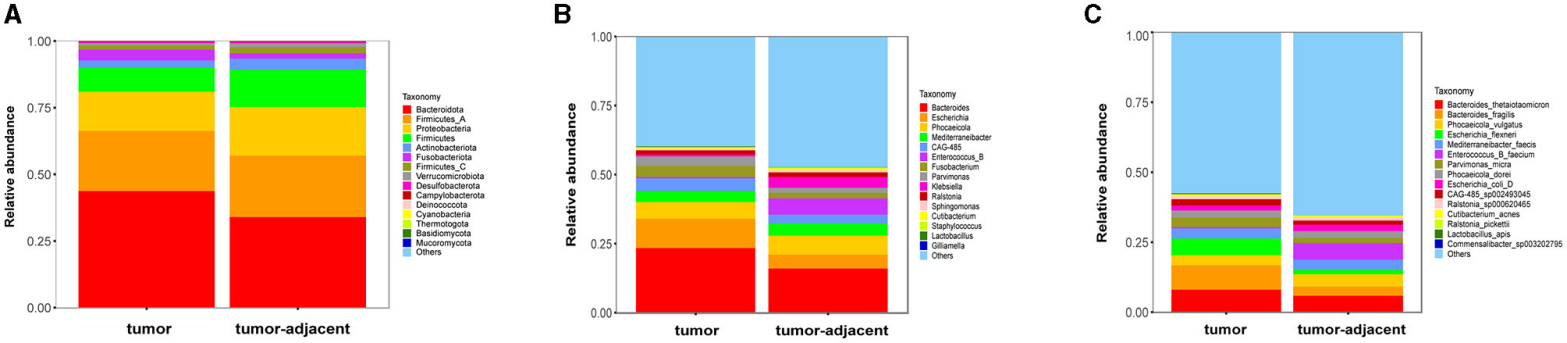
The relative abundance percentage of the top 15 bacteria in tumor- and tumor-adjacent tissues of CRC patients at phylum **(A)**, genus **(B)**, and species **(C)** level.

A total of 338,995,268 2bRAD-M bacterial gene reads with high quality were sequenced to determine the microbial compositions in 12 serum samples and 18 colorectal tissue samples. The average number of the generated sequence reads was 8,668,868 per serum sample, 12,665,054 per tumor sample, and 13,442,597 per tumor-adjacent tissue. After the sequence filtering, alignment and annotation, the relative abundance of top 15 bacterial community of each sample at phylum and species levels were shown in Figures 1A, B. We analyzed the relative abundance of the bacteria in serum and tissue samples at phylum level. It was found that the bacteria in serum mainly included Pseudomonadota, Bacillota, and Actinomycetota, while the microbiota in tissues was dominated by Bacteroidota, Bacillota*-A* and Pseudomonadota (Figure 1C). In addition, more than 50% of bacteria was composed of Pseudomonadota, Bacteroidota, and Bacillota phyla (serum = 80.85%, tissue = 66.90%). The relative abundance of Pseudomonadota and Bacillota phyla in serum samples was much higher than that in tissue samples, respectively (*P* < 0.01 for Bacillota; *P* < 0.0001 for Pseudomonadota), whereas the phylum Bacteroidota was the opposite (*P* < 0.0001; Figure 1D). At species level, the proportions of the top 15 species in serum and tissue samples only accounted for 46.34 and 38.49%, respectively (Figure 1E). Moreover, the relative abundance of the top 15 bacteria at phylum, genus and species level in tumor- and tumor-adjacent tissues was displayed in Figures 2A–C. No significant difference for the microbial community composition was observed between tumors and the tumor-adjacent tissues.

### 3.3 Serum microbiota diversity of CRC patients

**Figure 3:**
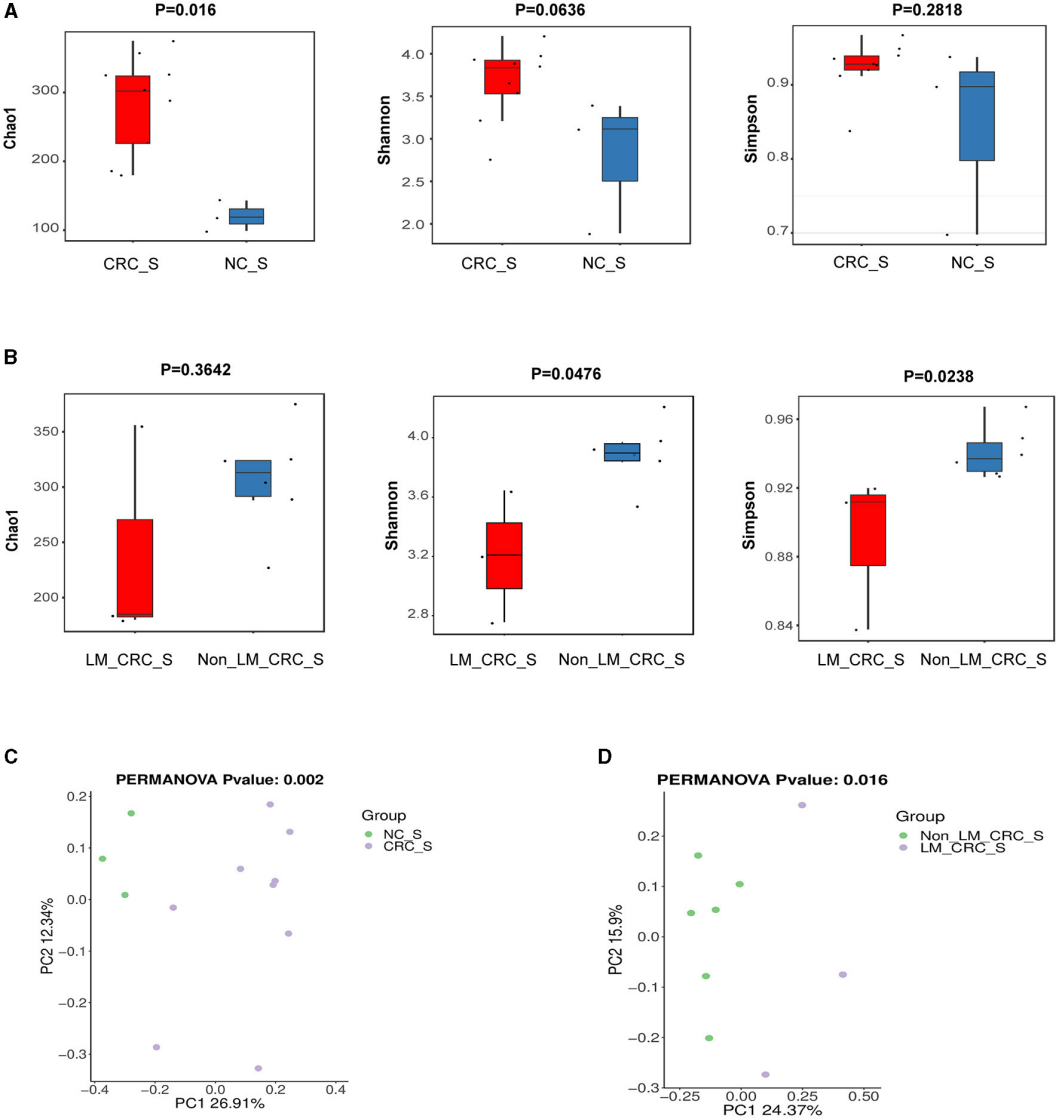
α-diversity and β-diversity analyses of serum microbiota. α-diversity of serum microbiota including Chao1, Shannon, and Simpson indexes, the control and CRC groups **(A)**, CRC with liver metastasis and CRC without liver metastasis group **(B)**; PCoA plot on β-diversity of serum microbiota, the control and CRC groups **(C)**, CRC with liver metastasis and CRC without liver metastasis group **(D)**.

**Figure 4:**
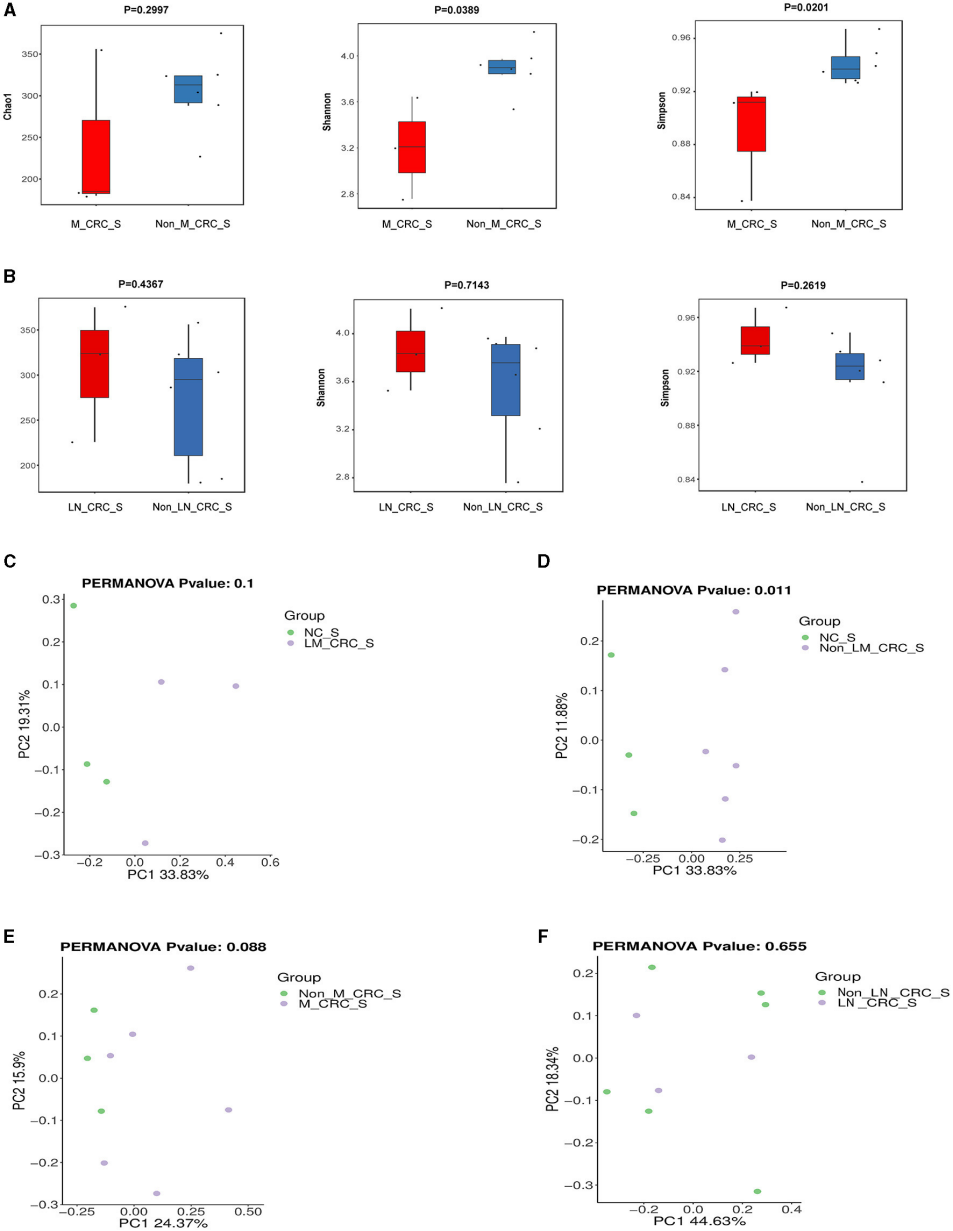
Diversity analysis of serum microbiota according to the status of metastasis. **(A)** α-diversity of serum microbiota including Chao1, Shannon, and Simpson indexes in CRC with and without metastasis. **(B)** α-diversity of serum microbiota including Chao1, Shannon, and Simpson indexes in CRC with and without lymph node metastasis. PCoA plot on β-diversity of serum microbiota between control and CRC with liver metastasis **(C)**, control and CRC without liver metastasis **(D)**, CRC with and without metastasis **(E)**. **(F)** PCoA plot on β-diversity of serum microbiota in CRC with and without lymph node metastasis.

The α-diversity of microbiota in serum between CRC group and healthy control group was assessed by Chao1, Shannon, and Simpson indexes (Figure 3A). The results showed that the α-diversity of serum microbiota in CRC patients was significantly increased compared to that in healthy controls, but only Chao1 index was statistically significant (*P* < 0.05). Considering the effect of gender on microbiota, we analyzed the α-diversity of serum microbiota in CRC group excluding male patients (Supplementary Figures S1A–C), and found the results were also significantly higher than healthy controls, so whether we exclude male patients or not, the results were the same. Interestingly, higher α-diversity of microbial communities was found in serum samples from CRC patients without liver metastasis than those with liver metastasis, as evidenced by Shannon index (*P* = 0.0476) and Simpson index (*P* = 0.0238) (Figure 3B). There was significant difference for α-diversity of the microbial community in serum of metastatic CRC patients compared to non-metastatic CRC patients, predicted by Chao1 (*P* > 0.05), Shannon (*P* < 0.05), and Simpson indexes (*P* < 0.05) (Figure 4A). Furthermore, no significance was found for microbial α-diversity in serum between lymph node metastasis and non-lymph node metastasis groups (Figure 4B). To estimate the differences of microbial communities in serum between groups, the β-diversity analysis was performed using principal-coordinate analysis (PCoA) based on the binary-jaccard distance. The results in Figures 3C, D showed that the differences of serum microbiota between CRC group and healthy control group (*R*^2^ = 0.205, *P* = 0.002), and between CRC with liver metastasis group and CRC without liver metastasis group (*R*^2^ = 0.217, *P* = 0.016) were both statistically significant. In addition, the PCoA result of serum microbiota between CRC group excluding male patients and healthy control group was different statistically (Supplementary Figure S1D). The β-diversity of serum microbiota was not different between CRC with liver metastasis and healthy control group (*R*^2^ = 0.280, *P* = 0.1) (Figure 4C). The β-diversity of serum microbiota from CRC patients without liver metastasis was significantly different from healthy controls (*R*^2^ = 0.33, *P* = 0.011) (Figure 4D). Furthermore, there was no difference for β-diversity of serum microbiota between metastatic CRC group and non-metastatic CRC group (*R*^2^ = 0.159, *P* = 0.088) (Figure 4E), as well as between the lymph node metastasis and non-lymph node metastasis groups (*R*^2^ = 0.093, *P* = 0.655) (Figure 4F). As a result, the analyses of α-diversity and β-diversity have suggested alterations in microbial community diversities in serum samples of CRC patients. In addition, the status of CRC progression, particularly liver metastasis status, might affect the diversities of serum microbiota.

### 3.4 Specific serum microbiota profile in CRC

**Figure 5:**
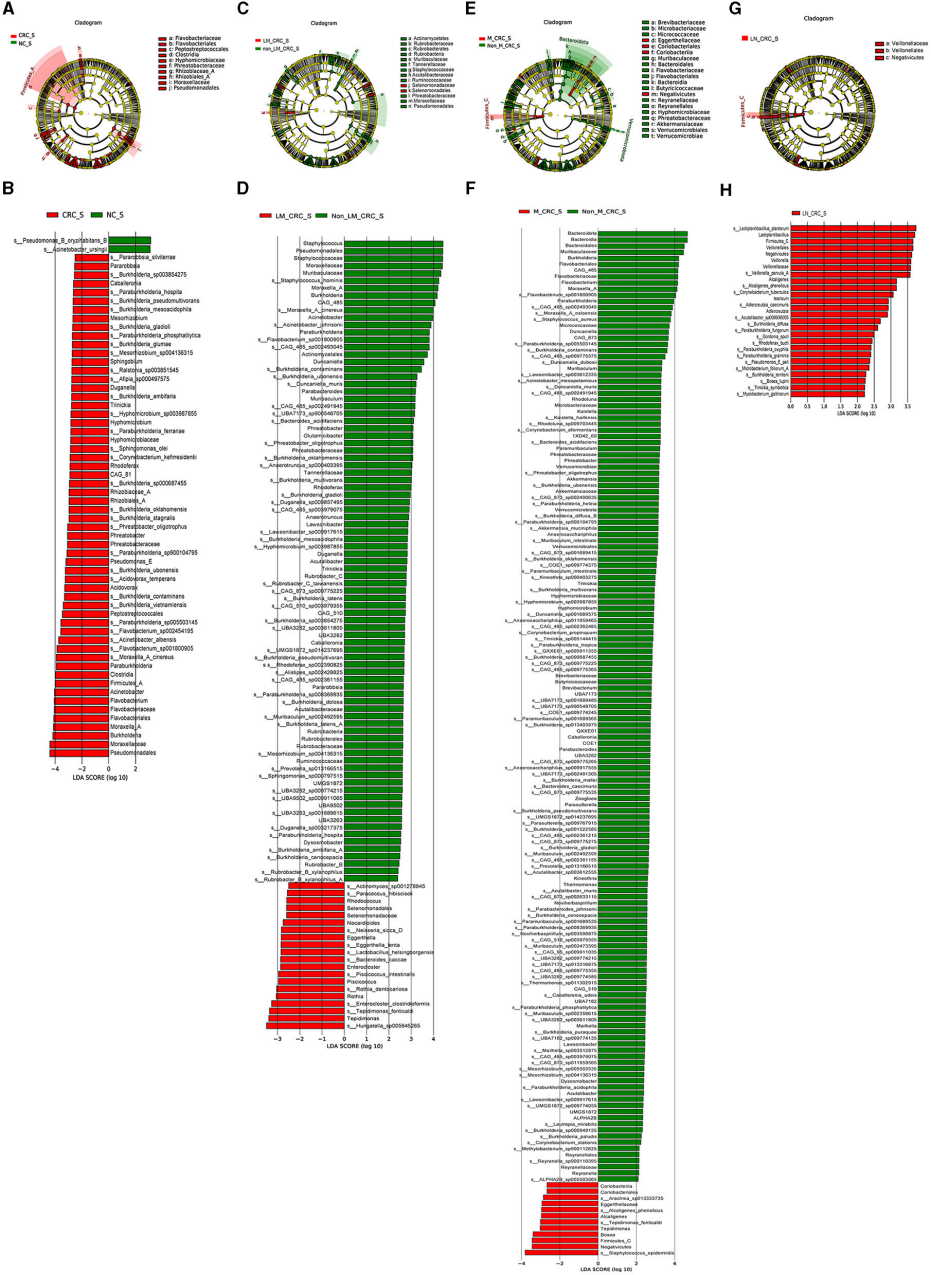
Specific serum microbiota profile in CRC predicting by LEfSe analysis. Cladograms showing the phylogenetic structure of serum bacterial communities from the healthy control and CRC group **(A)**, CRC with and without liver metastasis group **(C)**, CRC with and without metastasis **(E)**, CRC with and without lymph node metastasis **(G)**. Histograms of differentially abundant taxa between the healthy control and CRC group **(B)**, CRC with and without liver metastasis group **(D)**, CRC with and without metastasis **(F)**, CRC with and without lymph node metastasis **(H)**.

**Figure 6:**
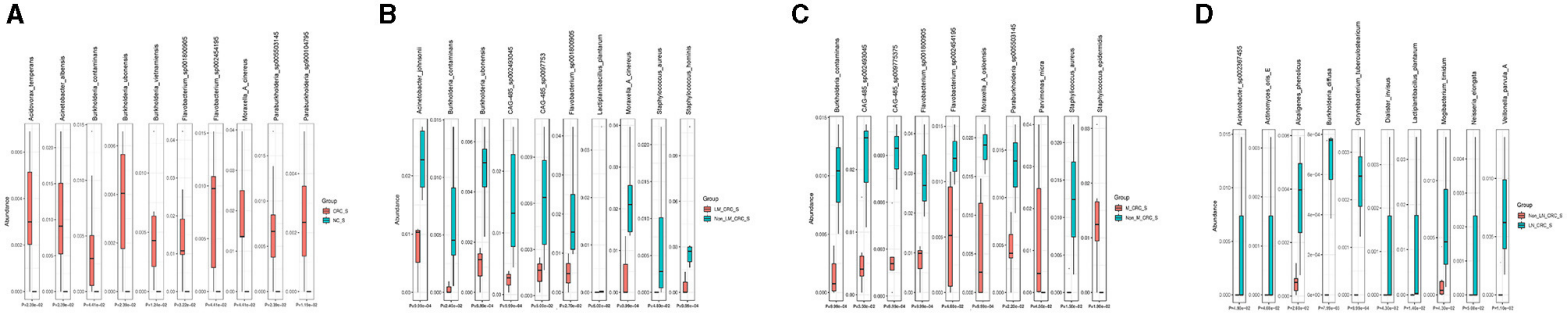
Boxplots on the top 10 differential species between control and CRC group **(A)**, CRC with and without liver metastasis **(B)**, CRC with and without lymph node metastasis **(C)**, and CRC with and without metastasis **(D)**.

The linear discriminant analysis effect size (LEfSe) analysis was conducted to investigate the specific microbiota profiles in serum of each group. A cladogram representing the phylogenetic structure and predominant bacteria in serum samples from the healthy controls and CRC patients is shown in Figure 5A. The LEfSe analysis had suggested a total of 60 distinguished taxa that were marked in serum samples from the two groups (CRC group and healthy control group) by LDA scores above 2.0. Among them, only 2 taxa were identified to be enriched in serum of the healthy control group, while the other 58 taxa were predominant in serum of the CRC group (Figure 5B), while 10 taxa were predominant in serum of the CRC group male patients excluded (Supplementary Figure S1E). The species of Pseudomonas B oryzihabitans B and *Acinetobacter ursingii* were significantly enriched in the control group compared to CRC group. Besides, the abundances of some species were much higher in serum samples from CRC patients. The top 10 specific microbial species in CRC serum were Moraxella A cinereus, Flavobacterium sp001800905, *Acinetobacter albensis*, Flavobacterium sp002454195, Paraburkholderia sp005503145, *Burkholderia vietnamiensis, Burkholderia contaminans, Burkholderia ubonensis, Acidovorax temperans*, and Paraburkholderia sp900104795 (Figure 6A). Based on the status of CRC progression, the cladogram presenting the markedly different bacterial taxa and the LEfSe analysis predicting the specific serum microbiota profiles of CRC patients with and without liver metastasis had also been exhibited in Figures 5C, D. The abundance of 20 bacterial taxa was increased significantly in serum of CRC patients with liver metastasis (Figure 5D). Moreover, the top 10 specifically decreased species in serum samples from CRC patients with liver metastasis were shown in Figure 6B. The specific serum microbiota profiles of CRC patients with lymph node metastasis were shown in Figure 6C. We also illustrated the cladogram and distinguished taxa of serum bacterial profile in metastatic CRC patients and non-metastatic CRC patients using the LEfSe analysis, the results indicated that the abundance of 12 differentially taxa was increased in metastatic CRC patients (Figures 5E, F). The serum bacterial profile of CRC patients with lymph node metastasis compared to non-lymph node metastatic CRC patients predicted by the cladogram and the LEfSe analysis is shown in Figures 5G, H. The abundance of 26 bacterial taxa was increased significantly in CRC patients with lymph node metastasis (Figure 5H). In addition, the top 10 differentially species of serum microbiota in metastatic CRC patients are displayed in Figure 6D. Accordingly, there were specific serum microbiota profiles in CRC considering the status of CRC progression. Those altered bacterial species might be useful biomarkers predicting the occurrence and progression of CRC.

### 3.5 Predicted functions and KEGG pathways of serum microbiota in CRC

**Figure 7:**
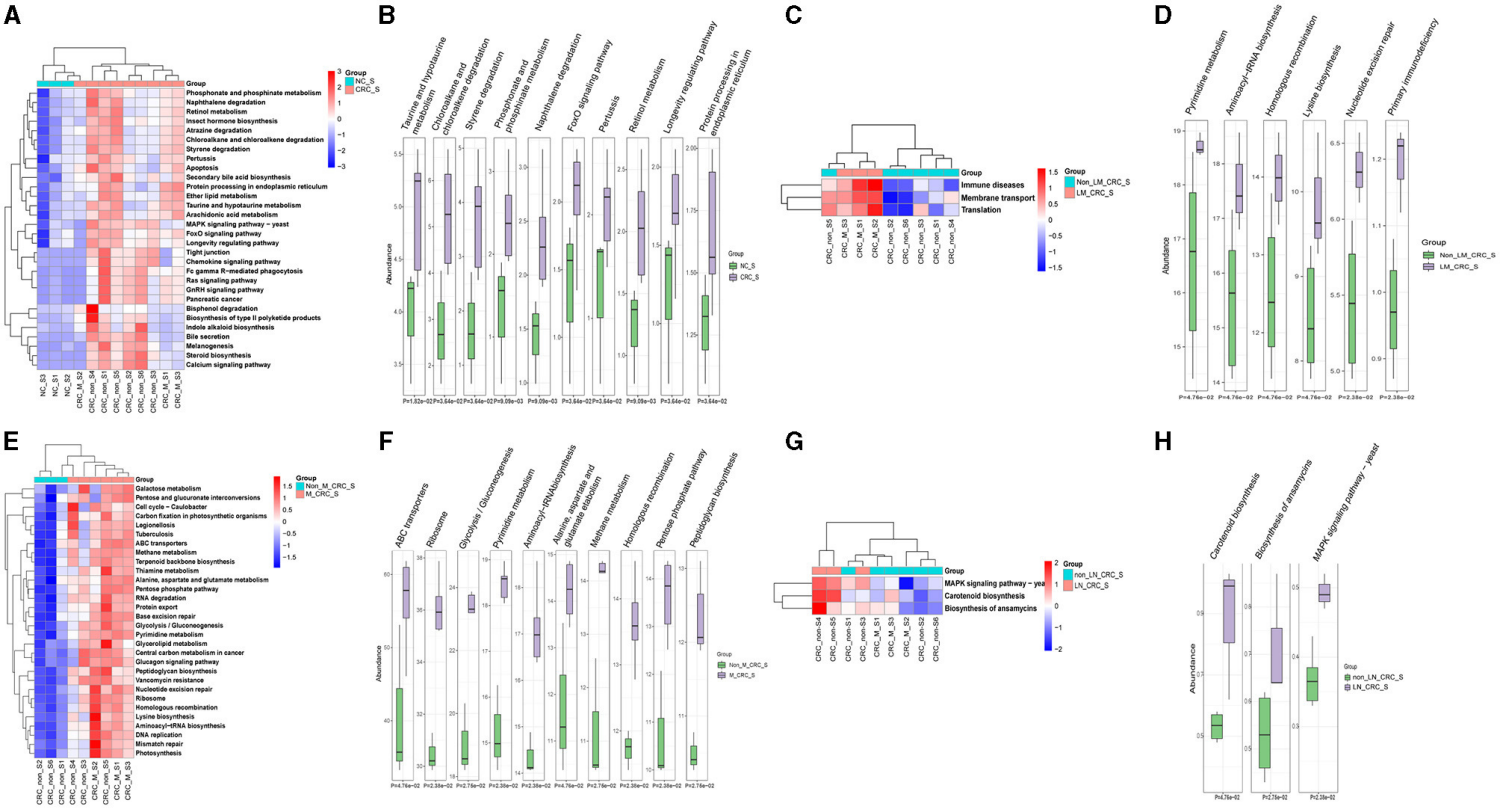
Predicted functions and pathways of serum microbiota in CRC. Heatmaps of predicted function in serum microbiota between the healthy control and CRC group **(A)**, CRC with and without liver metastasis group **(C)**, CRC with and without metastasis **(E)**, CRC with and without lymph node metastasis **(G)**. The top 10 differentially KEGG pathways between the healthy control and CRC group **(B)**, CRC with and without liver metastasis group **(D)**, CRC with and without metastasis **(F)**, CRC with and without lymph node metastasis **(H)**. *P*-value was calculated by Wilcoxon test.

The predicted KEGG pathways between the healthy control and CRC groups were analyzed using PICRUSt2 software. A total of 348 pathways were estimated regarding the potential role of serum microbiota in CRC, including cellular processes, environmental information processing, genetic information processing, human diseases, metabolism, and organismal systems. The heatmap analysis had implicated that the different relative abundance of serum microbiota in CRC was associated with different biological functions (Figure 7A). The predicted functions of altered serum microbiota in CRC were primarily enriched in taurine and hypotaurine metabolism, phosphonate and phosphinate metabolism, foxO signaling pathway, pertussis, retinol metabolism, longevity regulating pathway, and protein processing in endoplasmic reticulum (Figure 7B). Besides, compared to the group of CRC patients without liver metastasis, KEGG pathways in level1 of immune diseases, membrane transport, and translation function were predicted to be associated with alterations of serum microbiota in CRC with liver metastasis (Figure 7C). In details, the relative abundance of serum microbiota CRC with liver metastasis was mainly involved in regulating aminoacyl-tRNA biosynthesis, homologous recombination, mismatch repair, lysine biosynthesis, nucleotide excision repair, primary immunodeficiency, and proteoglycans in cancer (Figure 7D). The predicted KEGG pathways in metastatic CRC patients were different from CRC patients without metastasis (Figure 7E), and notably different KEGG pathways were enriched in CRC with metastasis group, such as ABC transporters, ribosome, glycolysis, pyrimidine metabolism, aminoacyl-tRNA biosynthesis (Figure 7F). Furthermore, the KEGG pathways predicted in lymph node-metastatic CRC patients were different from non-lymph node-metastatic CRC patients (Figure 7G). The significant KEGG pathways in lymph node-metastatic CRC patients mainly included carotenoid biosynthesis, ansamycins biosynthesis, MAPK signaling pathway (Figure 7H). Taken together, the serum microbiota profiles are specific in CRC, which might also affect the progression of CRC through the predicted pathways mentioned above.

## 4 Discussion

The screening and diagnosis of CRC mainly rely on imaging methods, mainly including barium meal enterography, spiral CT, colonoscopy, and ultrasound examination (Issaka et al., 2023). The detections of tumor-related antigens and tumor susceptible genes are also key approached for CRC screening and auxiliary diagnosis (Jayasinghe et al., 2023). The colonoscopy screening is a useful method in the early diagnosis of CRC, reducing the risk of cancer-related mortality (Doubeni et al., 2018; Bretthauer et al., 2022; Jayasinghe et al., 2023). However, colonoscopy is prone to missed diagnosis. Adenomas below 10 mm, atypical serrated polyps and Lipoma are the main reasons for missed diagnosis. Therefore, identifying more effective and non-invasive screening methods for CRC diagnosis remains an urgent clinical problem to be resolved. Non-invasive CRC screening methods have the advantages of painless, simple and fast, such as guaiac-based fecal occult blood tests (gFOBTs), fecal immunochemical tests (FITs), multi-target stool DNA (mt-sDNA) test, and serology detection of methylated septin 9 gene (Ladabaum et al., 2020). In recent years, the application of fecal microorganisms as a predictor of CRC has gradually risen (Abo-Hammam et al., 2024). The intricate relationship between the microbiome and CRC extends beyond the confines of the gut, encompassing systemic circulation and distant metastatic sites (Mignini et al., 2024). Understanding the distinct roles and dynamics of microbiota in the serum and gut microenvironments across different stages of CRC and metastatic dissemination is crucial for deciphering disease pathogenesis and tailoring therapeutic interventions. Beyond the gut, the systemic circulation acts as a conduit for microbial dissemination and immune modulation in CRC. Recent studies have highlighted the presence of a circulating microbiome, comprising microbial DNA, RNA, and extracellular vesicles (EVs) (Li et al., 2023; Yoon et al., 2023), which may reflect microbial translocation from the gut mucosa, tumor shedding areas to carcinogenic sites. Furthermore, alterations in microbial metabolites, such as short-chain fatty acids (SCFAs) and secondary bile acids, play pivotal roles in modulating tumor growth, angiogenesis, and immune evasion mechanisms in CRC (Cai et al., 2024; Cong et al., 2024). However, the CRC-specific microorganism in circulation has not been paid enough attention in the past few years.

Dysbiosis within the gut microbiome evolves throughout CRC progression, marked by shifts in microbial composition and function. This dysbiosis, characterized by altered diversity and an enrichment of pathogenic taxa, contributes to CRC initiation, promotion, and progression through complicated mechanisms, such as inflammation, immune dysregulation, and metabolic perturbations (Silva et al., 2021; Wang et al., 2023). In early-stage CRC, dysbiosis manifests as a reduction in beneficial commensal bacteria, such as *Bacteroides* and Bacillota, alongside an increase in pro-inflammatory and pathogenic taxa, such as *Fusobacterium nucleatum* and *Enterococcus faecalis* (Amini et al., 2022; Conde-Perez et al., 2024). The microbial imbalance leads to local inflammation, mucosal barrier disruption, and remodeling of the tumor microenvironment, thereby fostering tumorigenesis. In advanced stages of CRC, including lymph node or liver metastasis, microbial dysbiosis intensifies with distinct microbial signatures emerging within the tumor microenvironment and adjacent tissues (Mignini et al., 2024; Xia et al., 2024). *F. nucleatum* is a common periodontal pathogen and plays an important role in the development and metastasis of many cancer types (Parhi et al., 2020; McIlvanna et al., 2021; Li et al., 2022). *F. nucleatum* subspecies are associated with CRC, and *F. nucleatum* subsp. animalis strain has been found to co-aggregate with CRC-linked species, such as *Campylobacter concisus, Hungatella hathewayi*, and *Parvimonas micra* (Robinson and Allen-Vercoe, 2023). In this study, we have found the CRC-specific microbiome in serum using the 2bRAD-M sequencing method, although the abundance is much lower in the blood samples and tissues than the fecal samples. Notable difference in the microbial compositions and diversities was observed in serum samples from CRC patients but not in intestinal tissues. When comparing with the healthy controls, the serum microorganisms of CRC patients were lacking Pseudomonas B oryzihabitans B and *Acinetobacter ursingii*. However, a variety of microorganisms not found in serum samples from healthy individuals were confirmed in samples from CRC patients for the first time, such as Moraxella A cinereus, Flavobacterium sp001800905, *Acinetobacter albensis*, Flavobacterium sp002454195, Paraburkholderia sp005503145, *Burkholderia vietnamiensis, Burkholderia contaminans, Burkholderia ubonensis, Acidovorax temperans*, and Paraburkholderia sp900104795. There are few studies reporting these bacterial strains in CRC probably due to the limited abundance, which is too low that it is easy to be ignored in common detection except from 2bRAD-M sequencing. Compared to the non-liver metastatic CRC group, patients with liver metastatic CRC lacked many microorganisms in their serum, such as *Staphylococcus, Pseudomonadales*, Moraxella A, *Burkholderia*, CAG485*, Muribaculaceae*, and *Paraburkholderia*. It has been reported that *Streptococcus gallolyticus* subspecies gallolyticus had a strong association with CRC and promotes the progression of colon tumors (Kumar et al., 2022), while *Streptococcus epidermidis* was observed to enrich in metastatic CRC in our study. Besides, many microorganisms in the serum samples from patients with metastatic CRC were not detected in those from patients with non-metastatic CRC. The majority of them have not been previously reported but found to be rich in the peripheral circulation of liver metastatic CRC, such as Hungatella sp005845265, *Tepidamonas, Tepidamonas fonticaldi, Enterocloster clostridioformis, Rothia, Rothia dentocariosa, Piscicoccus, Piscicoccus intestinalis, Enterocloster*, and *Bacteroides caccae*. In previous study, the high abundance of *Hungatella hathewayi* was linked to the low survival rate of CRC (Huang et al., 2023), but *Hungatella* sp. subspecies in CRC have not been reported. *Rothia dentocariosa*, gram-positive bacteria, is generally considered to be the normal flora of oral cavity and respiratory tract (Tsuzukibashi et al., 2017). Other studies suggested that *R. dentocariosa* is not only related to periodontal inflammatory disease, but can also cause keratitis infective, endocarditis, even sepsis (Khan et al., 2014; Yeung et al., 2014; Elkattawy et al., 2021; Greve et al., 2021; Williams et al., 2021), while it may be a potential biomarker for liver metastatic CRC in this study. Furthermore, some specific serum microorganisms could be used to distinguish CRC with or without metastasis of lymph node or liver. Identifying combinations from these CRC-specific microorganisms as potential markers for CRC screening and risk stratification is worthy to be explored in more future studies. It is imperative to have a comprehensive comprehension of the differential strains associated with disease at the species or strain level. The biological functions of bacterial strains, such as *F. nucleatum* and *R. dentocariosa*, are not straightforward or universally conserved. In addition to bacterial abundance and colonization, the diversity of bacterial strains beyond the species level may be underestimated due to single nucleotide polymorphisms (SNPs) or gene variations. The inability to achieve precise results at the strain level due to limitations in sequencing technology represents a significant limitation of our research.

The strongest differences among significantly differentially abundant KEGG pathways involved in the tumorigenesis of CRC included taurine and hypotaurine metabolism, chloroalkane and chloroalkene degradation, styerene degradation, phosphonate and phosphinate metabolism, Forkhead box O (FoxO) signaling pathway, retinol metabolism, and longevity regulating pathway. Taurine is a widely existing amino acid in human body. Metabolism of taurine conjugated bile acids by gut microbes generates hydrogen sulfide, a genotoxic compound (Ridlon et al., 2016; Hou et al., 2021). The reduction of intestinal bacteria *Hungatella hathewayi* can lead to the reduction of taurine levels in the blood (Li et al., 2020). We performed taurine ELISA assay for validation serum samples, and the result was showed that the taurine concentration of serum in CRC group was higher than control group, illustrated in Supplementary Figure S1F, which was consistent with the predicted functions of altered serum microbiota in CRC (Figure 7B). In our research, Hungatella sp005845265 was detected in serum samples from metastatic CRC patients, which can be used as a CRC-specific marker. Bisphosphonates are commonly used in the treatment of osteoporosis and metastatic bone cancer, which have also been proven to reduce the recurrence risk and mortality of invasive breast cancer (Mbese and Aderibigbe, 2021). It has been well-established that oral administration of bisphosphonates can effectively reduce the risk of CRC (Thosani et al., 2013; Vogtmann et al., 2017). The FoxO belongs to the fork head transcription factor family playing important roles in deciding cell fate decisions. FoxO serves as a tumor suppressor in a wide range of cancers (Farhan et al., 2017). Retinol and the retinol metabolism-related genes and enzymes participate in the tumorigenesis of CRC by regulating the function of colorectal cells (Sun et al., 2021). CRC patients exhibit retinoic acid synthesis defects, while retinoic acid supplementation significantly inhibited tumor growth in a CRC mouse model (Sun et al., 2021). Compared with non-metastatic patients, the predicted pathway of metastatic CRC patients was enriched in protein synthesis and DNA synthesis pathway. Accordingly, those predicted KEGG pathways and functional terms are highly predicted in the initiation and progression of CRC.

There are some limitations in the present study. The sample size in this study is too small to draw a more convincing conclusion, particularly regarding the estimation of CRC-specific microbiomes in peripheral circulation and cancer tissues according to the status of metastasis. Whether differential microorganisms can be used as markers for the screening and risk stratification needs to be verified in the future. The precise roles of the predicted functional terms and metabolic pathways in the pathogenesis and the progression of CRC warrant to be further investigated in more future research.

## 5 Conclusion

Microorganisms in cancer tissues are being explored by researchers. But serum microbiota is still a blind spot. In this study, we investigated the circulating microbiota at the species level between CRC patients and healthy individuals, and CRC patients with or without lymph node and liver metastases. Many differential and CRC-specific microorganisms have been confirmed in circulation and intestinal tissues by 2bRAD-M sequencing. Importantly, some of these well-documented microorganisms with low abundance in the gut microbiota have been demonstrated to be closely related to CRC onset and progression. All the findings have shed some light on the multifaceted interplay between microbiota and CRC, thereby providing critical microbial markers for CRC diagnosis, treatment, and prognostic assessment. Unraveling the microbial signatures and the underlying regulatory mechanisms in CRC holds transformative potentials in refining diagnostic ways, enhancing diagnostic sensitivity, and ushering the development of personalized CRC screening strategies.

## Data Availability

The datasets presented in this study can be found in online repositories. The names of the repository/repositories and accession number(s) can be found below: www.biosino.org/node, OEP004048.

## Supplementary material

Supplementary Figure S1 α-diversity of serum microbiota including Chao1 **(A)**, Shannon **(B)**, and Simpson **(C)** indexes in the healthy control and CRC group excluded male patients. **(D)** PCoA plot on β-diversity of serum microbiota in the healthy control and CRC group excluded male patients. **(E)** Histograms of differentially abundant taxa between the healthy control and CRC group excluded male patients. **(F)** The taurine concentration of serum in CRC group in the healthy control and CRC group. Student's *t*-test is performed to determine the differences between serum and tissue groups. ^*^*P* < 0.05.

Supplementary Table S1 The basic information of validation cohort.
