# Supplementary figures and images for: Colorectal cancer-specific microbiome in peripheral circulation and cancer tissues
Source: Front Microbiol. 2024 Aug 21;15:1422536. doi: 10.3389/fmicb.2024.1422536 (PMC11371800; doi:10.3389/fmicb.2024.1422536)

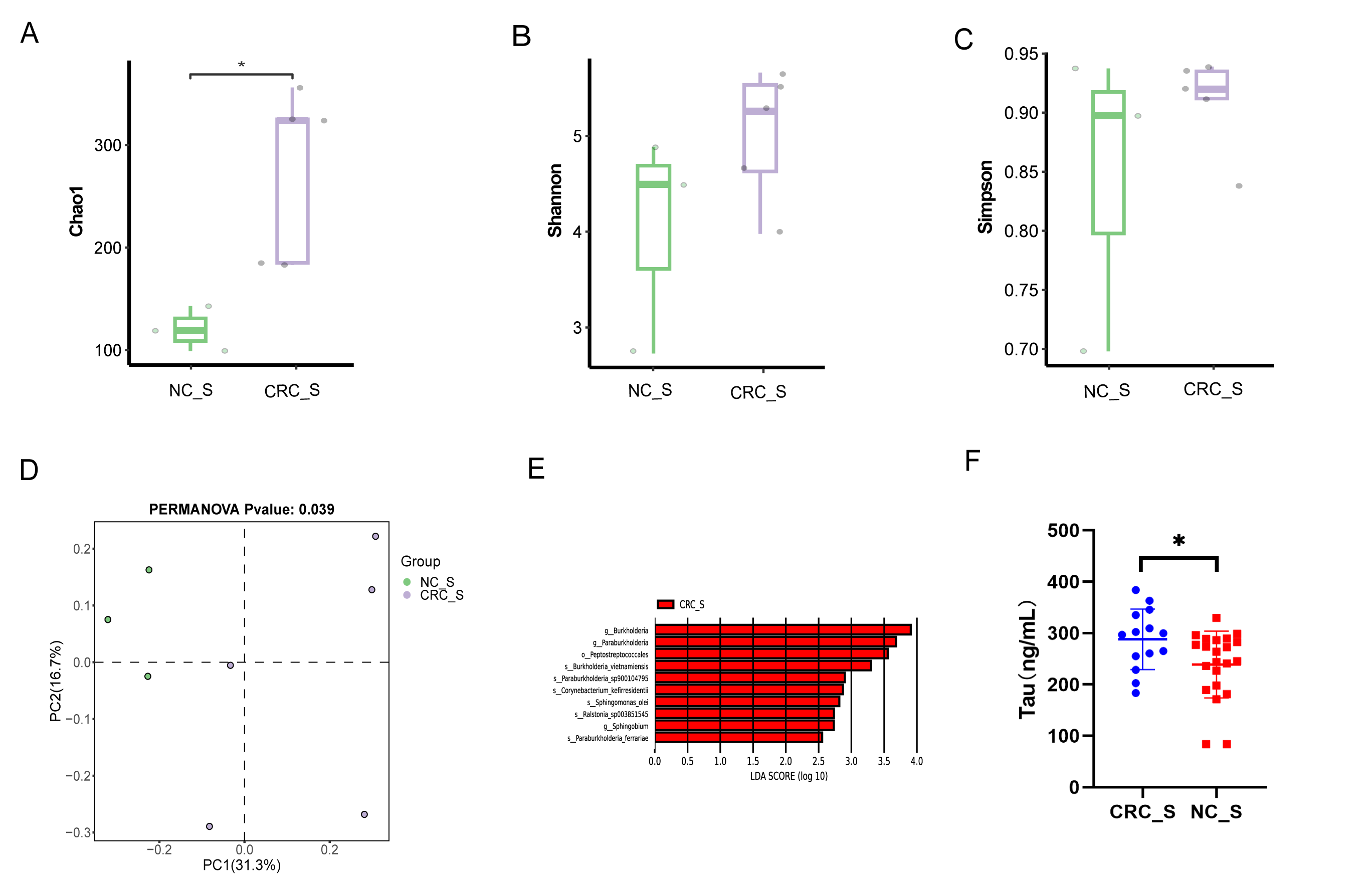

Supplement: Supplementary Figure S1 — α-diversity of serum microbiota including Chao1 (A), Shannon (B), and Simpson (C) indexes in the healthy control and CRC group excluded male patients. (D) PCoA plot on β-diversity of serum microbiota in the healthy control and CRC group excluded male patients. (E) Histograms of differentially abundant taxa between the healthy control and CRC group excluded male patients. (F) The taurine concentration of serum in CRC group in the healthy control and CRC group. Student's t-test is performed to determine the differences between serum and tissue groups. *P < 0.05. [file Image_1.TIF]
