# Supplementary material for: Colorectal cancer-specific microbiome in peripheral circulation and cancer tissues
Source: Front Microbiol. 2024 Aug 21;15:1422536. doi: 10.3389/fmicb.2024.1422536 (PMC11371800; doi:10.3389/fmicb.2024.1422536)
Supplement: Supplementary Table S1 — The basic information of validation cohort. [file Table_1.DOCX]

**Table S1. The basic information of validation cohort**

| Characteristics | CRC(n=14) | Control (n=22) |
| --- | --- | --- |
| Age | 65.6±10.8 | 65.4±4.1 |
| Male | 8 (57%) | 10 (45%) |
| Female | 6 (43%) | 12 (55%) |
| Annotation: the statistical significance for age is No. | | |
